# Cystic neuroendocrine tumor of the pancreas a challenging clinical diagnosis!

**DOI:** 10.11604/pamj.2019.33.129.19375

**Published:** 2019-06-21

**Authors:** Faten Limaiem

**Affiliations:** 1 University of Tunis El Manar, Tunis Faculty of Medicine, Tunis, Tunisia

**Keywords:** Pathology, pancreas, immunohistochemistry, neuroendocrine tumor

## Image in medicine

Cystic pancreatic neuroendocrine tumor accounts for 13-17% of pancreatic neuroendocrine tumors and 9-10% of resected cystic tumors. This unusual variant can make clinical diagnosis particularly challenging. A 28-year-old female patient presented with abdominal pain and dyspepsia. General and physical examination was unremarkable. On computed tomography scan a 7.5×5.4×5.2cm thick-walled cystic lesion was noted in the tail region of the pancreas in the stomach bed (A). On the basis of the above findings, the radiologist reported it as suggestive of solid pseudopapillary neoplasm and advised pathological correlation. No abdominal lymphadenopathy was noted. The patient underwent laparoscopic removal of a pancreatic cyst. Intraoperatively, a large cyst was seen located posterior to the stomach in retroperitoneum and was intimately related to the tail of the pancreas. The cyst could be enucleated. On histopathological examination, the sections from the cyst wall showed collagen-rich fibrous tissue in which were embedded small clusters and nests of round cells (B). The cells were monotonous, small to medium in size with amphophilic cytoplasm with indistinct cell borders. The nucleus showed typical stippled nuclear chromatin and no mitoses were noted (C). There was no capsular invasion. Neuroendocrine nature of these round cells was confirmed by performing immunohistochemistry with synaptophysin and chromogranin A (C). Ki67 stained <3% of the cells. These findings corroborated the benign nature of the disease indicating a very good prognosis. Postoperative recovery of the patient was uneventful

**Figure 1:**
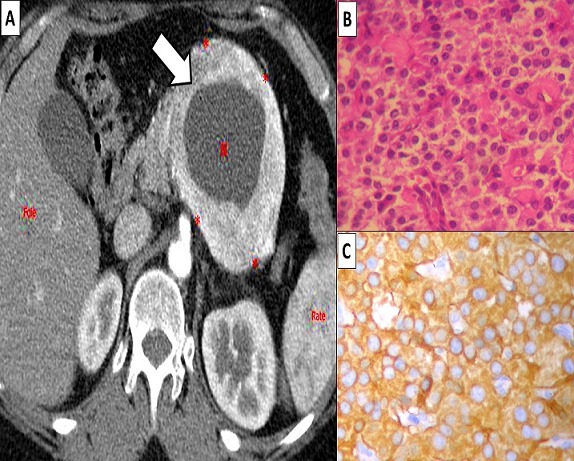
(A) computed tomography scan of the abdomen showing 6.5×5.4×5.2cm thick-walled cystic lesion in the tail of pancreas (arrow); (B) the tumor cells were monotonous, small to medium in size with amphophilic cytoplasm with indistinct cell borders. The nucleus showed typical stippled nuclear chromatin and no mitoses were noted (Hematoxylin and esosin ×100); (C)immunohistochemical staining of the tumor cells showing positivity for chromogranin A (Immunohistochemistry, ×100)

